# Supplementary material for: Kinetics of the Cell Cycle Arrest Biomarkers (TIMP2 and IGFBP7) for the Diagnosis of Acute Kidney Injury in Critically Ill COVID-19 Patients
Source: Diagnostics (Basel). 2023 Jan 15;13(2):317. doi: 10.3390/diagnostics13020317 (PMC9857893; doi:10.3390/diagnostics13020317)
Supplement: Supplementary file 1 [file diagnostics-13-00317-s001.zip › diagnostics-2081728-supplementary.pdf]

Article

Kinetics of the Cell Cycle Arrest Biomarkers (TIMP2 and IGFBP7) for the Diagnosis of Acute Kidney Injury in Critically Ill COVID-19 Patients

Figure S1:Correlation between IL-6 and TIMP2-IGBP7 values at different time points

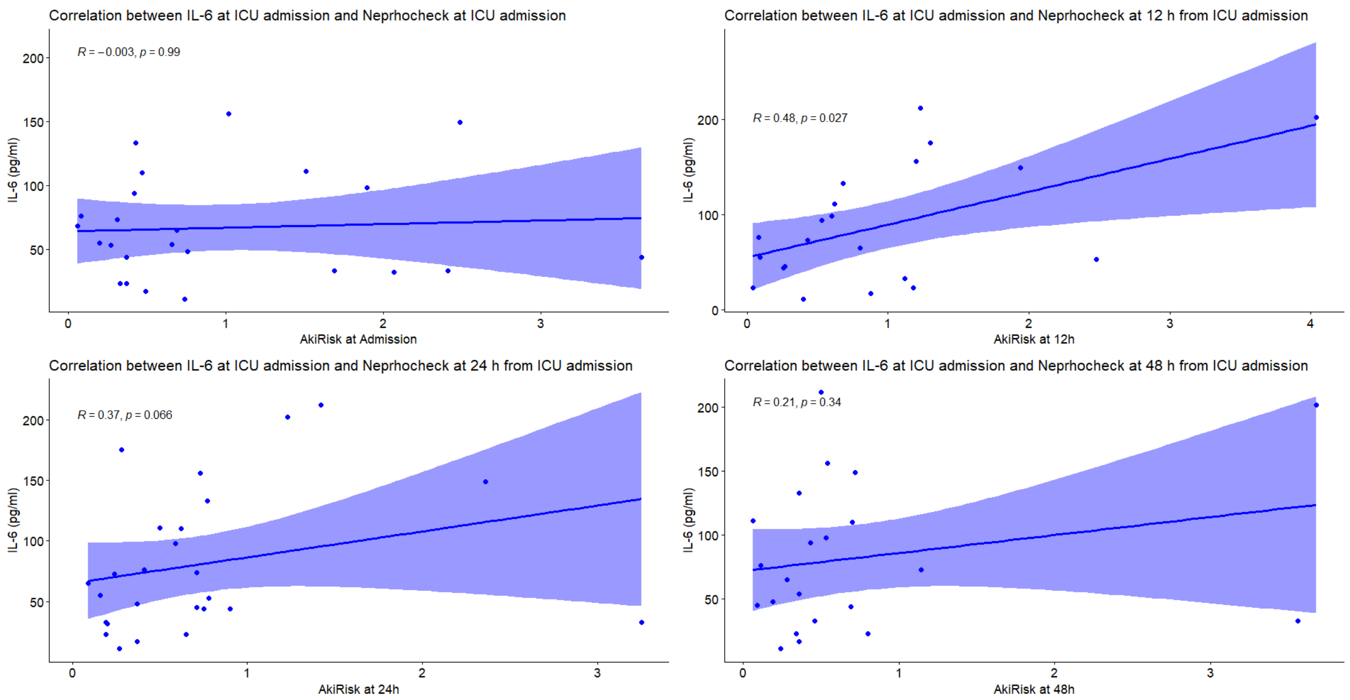

Figure S2:Correlation between highest IL-6 and TIMP2-IGBP7 values at different time points

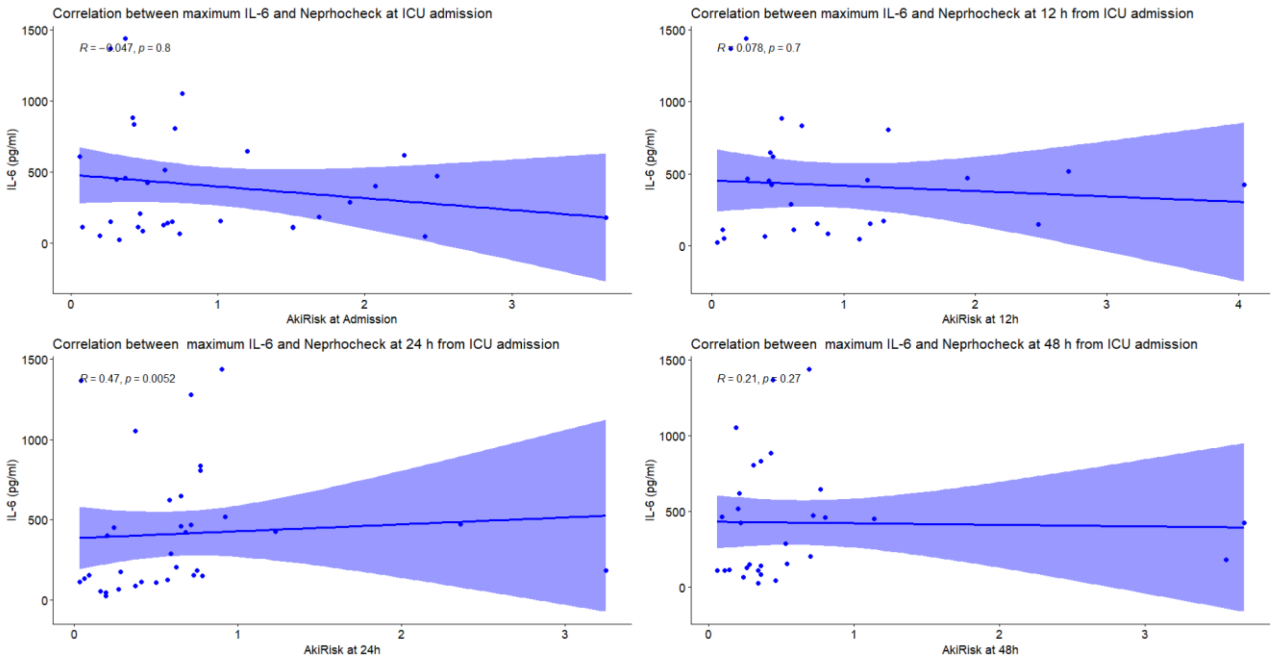

Figure S3: ROC curves of AKIRisk at 12h vs Serum Creatinine (sCR) at ICU admission, vs combination of both markers

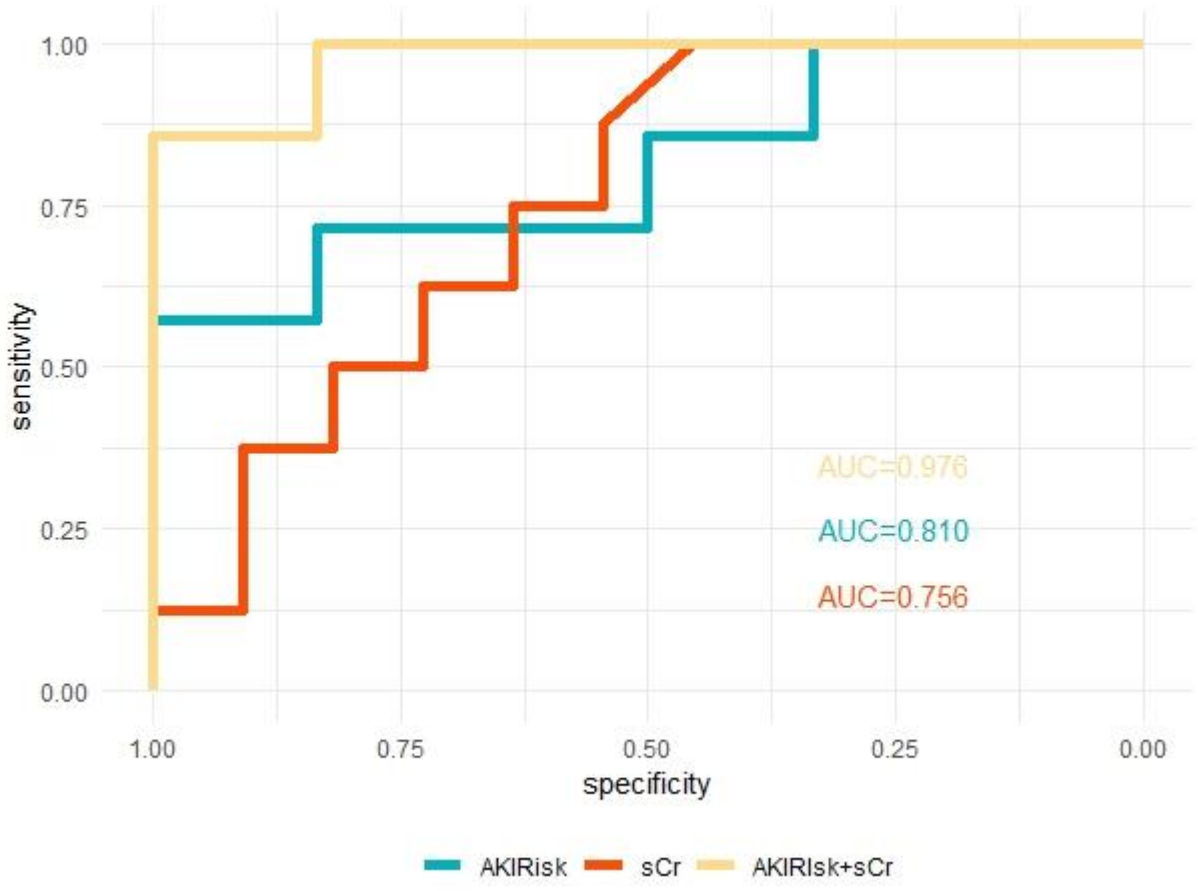

Table S1: Laboratory data (means, standard errors) in all patients, and divided by severity of AKI

Characteristic	N	Overall, N = 411	No or Mild AKI, N = 131	Moderate AKI, N = 191	Severe AKI, N = 91
sCr at ER admission (µmol/L)	36	92 (5)	84 (10)	93 (7)	101 (10)
sCr at ICU admission (µmol/L)	41	77 (4)	71 (7)	75 (6)	88 (9)
sCr Maximum value (µmol/L)	41	130 (16)	114 (24))	103 (7)	212 (54)
IL-6 at ICU admission (pg/ml)	29	80 (10)	80 (21))	71 (15)	98 (22)
IL-6 at ER admission (pg/ml)	30	76 (9)	44 (6))	78 (15)	96 (13)
IL-6 Maximum value (pg/ml)	39	408 (61)	417 (137)	355 (81)	503 (114)
AKI risk score at Time 0	34	0.94 (0.15)	0.96 (0.23)	1.00 (0.25)	0.79 (0.25)
AKI risk score at 12 hours	29	0.92 (0.17)	0.47 (0.13)	0.81 (0.18)	1.65 (0.49)
AKI risk score at 24 hours	36	0.65 (0.11)	0.81 (0.27)	0.47 (0.07)	0.80 (0.26)
AKI risk score at 48hours	32	0.60 (0.15)	0.71 (0.37)	0.39 (0.05)	0.88 (0.42)
